# Phenotypic and molecular spectrum of pyridoxamine‐5′‐phosphate oxidase deficiency: A scoping review of 87 cases of pyridoxamine‐5′‐phosphate oxidase deficiency

**DOI:** 10.1111/cge.13843

**Published:** 2020-09-16

**Authors:** Malak Alghamdi, Fahad A. Bashiri, Marwa Abdelhakim, Nouran Adly, Dima Z. Jamjoom, Khalid M. Sumaily, Bandar Alghanem, Stefan T. Arold

**Affiliations:** ^1^ Medical Genetics Division, Department of Pediatrics, College of Medicine King Saud University Riyadh Saudi Arabia; ^2^ Department of Pediatrics King Saud University Medical City Riyadh Saudi Arabia; ^3^ Neurology division, Department of Pediatrics, College of Medicine King Saud University Riyadh Saudi Arabia; ^4^ Computer, Electrical and Mathematical Science and Engineering Division (CEMSE), Computational Bioscience Research Center (CBRC) King Abdullah University of Science and Technology (KAUST) Thuwal Saudi Arabia; ^5^ College of Medicine Research Center, College of Medicine King Saud University Riyadh Saudi Arabia; ^6^ Department of Radiology and Medical Imaging, College of Medicine King Saud University Riyadh Saudi Arabia; ^7^ Clinical Biochemistry Unit, Department of Laboratory Medicine King Saud University Medical City, King Saud University Riyadh Saudi Arabia; ^8^ Medical Research Core Facility and Platforms (MRCFP) King Abdullah International Medical, Research Center/King Saud bin Abdulaziz University for Health Sciences (KSAU‐HS), King, Abdulaziz Medical City (KAMC), NGHA Riyadh Saudi Arabia; ^9^ Computational Bioscience, Research Center (CBRC); Division of Biological and Environmental Sciences and Engineering, (BESE) King Abdullah University of Science and Technology (KAUST) Thuwal Saudi Arabia; ^10^ Centre de Biochimie Structurale, CNRS, INSERM Université de Montpellier Montpellier France

**Keywords:** neonatal epileptic encephalopathy, PLP, PNPO, seizures, vitamin‐response epilepsy

## Abstract

Pyridoxamine‐5′‐phosphate oxidase (PNPO) deficiency is an autosomal recessive pyridoxal 5′‐phosphate (PLP)‐vitamin‐responsive epileptic encephalopathy. The emerging feature of PNPO deficiency is the occurrence of refractory seizures in the first year of life. Pre‐maturity and fetal distress, combined with neonatal seizures, are other associated key characteristics. The phenotype results from a dependency of PLP which regulates several enzymes in the body. We present the phenotypic and genotypic spectrum of (PNPO) deficiency based on a literature review (2002‐2020) of reports (n = 33) of patients with confirmed PNPO deficiency (n = 87). All patients who received PLP (n = 36) showed a clinical response, with a complete dramatic PLP response with seizure cessation observed in 61% of patients. In spite of effective seizure control with PLP, approximately 56% of patients affected with PLP‐dependent epilepsy suffer developmental delay/intellectual disability. There is no diagnostic biomarker, and molecular testing required for diagnosis. However, we noted that cerebrospinal fluid (CSF) PLP was low in 81%, CSF glycine was high in 80% and urinary vanillactic acid was high in 91% of the cases. We observed only a weak correlation between the severity of PNPO protein disruption and disease outcomes, indicating the importance of other factors, including seizure onset and time of therapy initiation. We found that pre‐maturity, the delay in initiation of PLP therapy and early onset of seizures correlate with a poor neurocognitive outcome. Given the amenability of PNPO to PLP therapy for seizure control, early diagnosis is essential.

## Graphical abstract

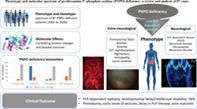

## 1 INTRODUCTION

Pyridoxamine‐5′‐phosphate oxidase (PNPO) deficiency is a recently recognized autosomal recessive neonatal epileptic encephalopathy (MIM#610090). This deficiency manifests within hours of birth as a severe seizure disorder that does not respond to anticonvulsant drugs and can be fatal, if untreated. Seizures can cease with the administration of the active form of B6 pyridoxal 5′‐phosphate (PLP) but are not always responsive to pyridoxine (PN).^1^

The first case was identified based on a clinical and biochemical profile that was consistent with PLP‐responsive neonatal epileptic encephalopathy.^2^ Later, Mills et al^1^ pinpointed that this condition is due to PNPO deficiency and the *PNPO* gene is the underlying genetic defect. Since the initial report, more than 30 studies have reported 87 cases.

PNPO uses flavin mononucleotide (FMN) as a cofactor to catalyze the oxidation of pyridoxine 5′‐phosphate (PNP) to PLP. Dysfunctional variants of *PNPO* result in an inability to catalyze the production of PLP. As a result, the body suffers from a scarcity of the active form of vitamin B6. PLP works as a cofactor for over 140 enzymes, representing every major class of enzymes except ligases^3^ (Supplementary Table S1). PLP‐dependent enzymes have essential roles in a variety of biochemical processes, including amino acid metabolism, glycolysis, gluconeogenesis, glycogenolysis, transsulfuration, polyamine biosynthesis, and synthesis of sphingoid bases, and the heme precursor δ‐aminolevulinic acid.^4^, ^5^ Hence, PLP is one of the most central molecules for the general cellular metabolism.

**FIGURE 1:**
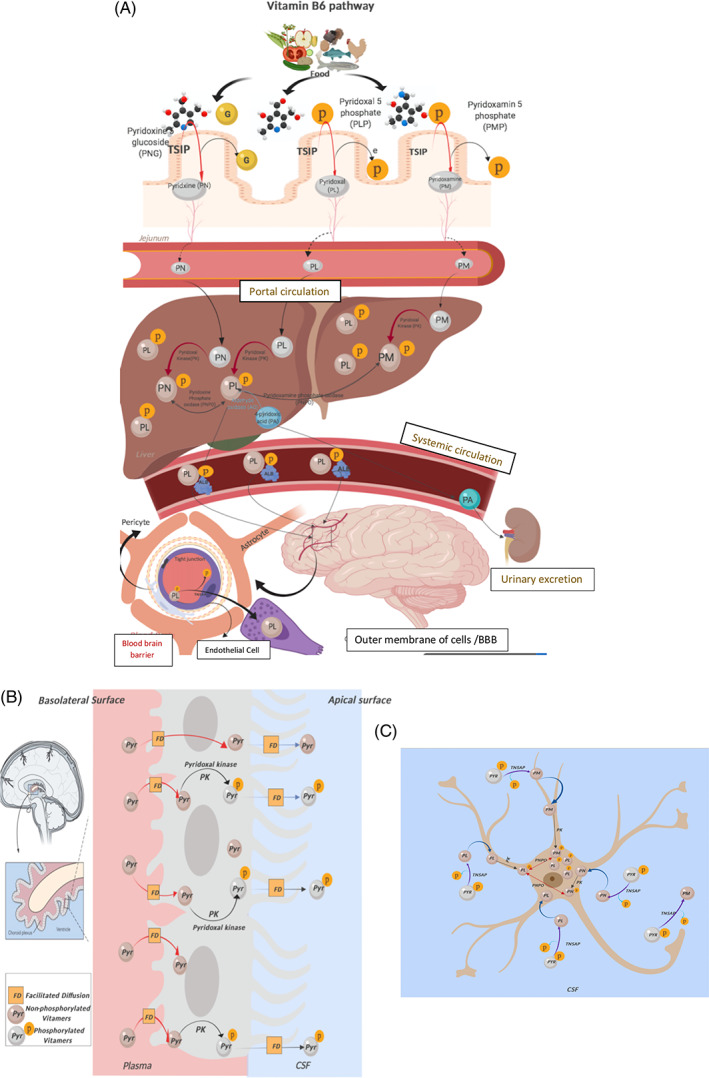
Vitamin B6 metabolism as it travels from the intestine to the portal circulation, crosses the blood‐brain barrier, and enters the brain cells. A, In the intestine, the dietary phosphorylated form is hydrolyzed to the free form by intestinal hydrolase (IH)/tissue‐specific intestinal phosphatase (TSIP) prior to absorption. This is followed by its uptake by intestinal cells, which is believed to occur through simple diffusion. Through portal circulation, the free B6 forms reach the liver, where metabolism in the liver is catalyzed by many enzymes. (1) Pyridoxal kinase (PK), (2) pyridoxal phosphate phosphatase, (3) pyridox(am)ine‐5′‐phosphate oxidase. B, The unphosphorylated forms of vitamin B6 are able to cross the blood‐brain barrier, probably by facilitated diffusion, mostly at the choroid plexus (CP). The CP traps PLP via pyridoxal kinase and can release PLP to a remarkable extent (and pyridoxal to a lesser extent). C, Excessive PLP in the CSF and extracellular space enters brain cells, and the B6 vitamers must be dephosphorylated so that they can enter brain cells and then metabolically trapped by being rephosphorylated by pyridoxal kinase. Pyridoxine phosphate and pyridoxamine phosphate are then oxidized by PNPO to form the active cofactor, PLP. PLP, pyridoxal 5′‐phosphate

Its metabolism in the liver requires many enzymes, including (1) pyridoxal kinase, which is responsible for vitamin B6 phosphorylation, an important step for the transfer of vitamin B6 to pyridoxal‐5‐phosphate; (2) pyridoxal phosphate phosphatase, the enzyme that is involved in the preferred degradation route of PLP by aldehyde oxidase to 4‐pyridoxic acid through PLP dephosphorylation; and (3) PNPO then catalyzes the last step in PLP synthesis^6^ (Figure 1).

Given the variety of PLP‐dependent enzymes, PLP deficiency might be expected to have diverse clinical presentations. However, the neurological phenotype is the predominant phenotype of PNPO deficiency, sometimes co‐occurring with non‐neurological manifestations such as impaired growth and hypochromic microcytic anemia that responds dramatically to treatment with PLP. Epileptiform convulsions in infants are a common presentation due to defective conversion of glutamic acid into γ‐aminobutyric acid (GABA). Other neurological manifestations including irritability and peripheral neuritis arise due to improper production of serotonin, epinephrine, norepinephrine, and GABA. Defects in the synthesis of sphingolipids lead to nerve demyelination, which is manifested as neuropathy.^4^, ^5^

This article reviews 87 cases of PNPO deficiency describing the spectrum of the neurological and non‐neurological phenotypes of PNPO deficiency as well as its diagnostic biochemical profile, genotypic basis, and therapeutic response to PLP and PN.

## 2 METHODS

For the literature review, we searched PubMed and Google scholar (http://www.ncbi.nlm.nih.gov/pubmed and https://scholar.google.com; 2002‐2020) using a combination of the following terms (restricted to humans): PNPO, pyridox(am)ine 5′ phosphate oxidase, PNPO, PLP‐dependent seizures, PLP‐responsive seizures. We selected all articles that met the following criteria: (1) published after the first case of PLP‐dependent seizure was reported in 2002; (2) published in English; and (3) reporting one or more PLP‐dependent epilepsy patients with confirmed PNPO deficiency, including a description of the clinical symptoms.

We extracted the clinical phenotypes, biochemical profiles, electroencephalography (EEG) results, and neuroimaging features from all the reviewed articles and all published data compiled them in one table (Suppl. Table S2). We studied the molecular effect of these variants on the PNPO structure and function, and correlated this information with the disease outcome. Other factors were included to predict outcomes, including pre‐maturity, fetal distress, seizure onset and time of initiation of PLP therapy.

## 3 RESULTS

### 3.1 Clinical aspect of PNPO Deficiency

The clinical data of 33 papers presented in the literature were collected and studied, for a total of 87 patients. Among the 87 cases, gender was identified in only 46 patients: 25 males and 21 females. Consanguinity was found in 25 patients and 24 cases had a family history of previously affected siblings or an early death with an undiagnosed condition manifested with refractory seizures (Supplementary Table S2). 22% of the patients (n = 19) died in the first year of life, one of them only after introduction of PLP. We have classified the clinical manifestations as neurological or extraneurological. The classical presentation is a neonatal epileptic encephalopathy, which is unresponsive to conventional anticonvulsant drugs and also to PN sometimes.

#### 3.1.1 Neurological manifestations of PNPO deficiency

**FIGURE 2:**
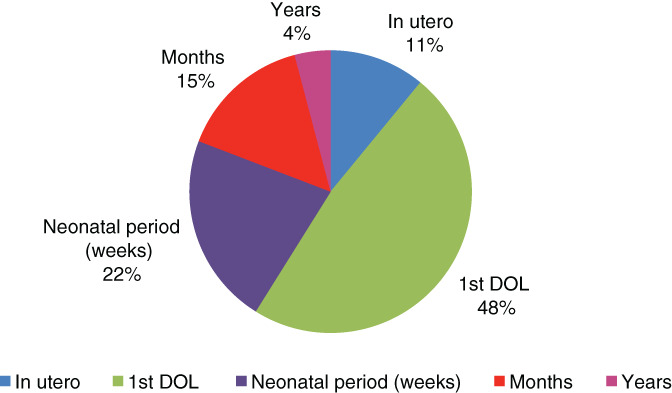
Seizure onset data for 87 reported cases with PNPO deficiency. PNPO, pyridoxamine‐5′‐phosphate oxidase

The described neurological changes varied widely among the literature. The most prominent neurological manifestation is a seizure within the first day of life, occurring in 59% of the reported cases. Abnormal fetal movement was noticed by the mother in the third trimester in 11% of the patients who have shown seizure within the first day of life (Figure 2).

Different types of clinical seizures have been observed in patients with PLP‐dependent epilepsy. The classical seizure pattern consists of prolonged seizures and recurrent episodes of status epilepticus or breakthrough seizures controlled with PN and/or PLP. 80% of the cases showed clonic seizures (focal and multifocal), tonic‐clonic seizures, generalized seizures, abnormal movement, or a combination of two or more of those types in the same episode or in alternation between episodes; absence/staring episodes occurred in 7% of cases.^7^, ^8^ Furthermore, paroxysmal movement disorder was observed in 10% of cases.^9^ Abnormal eye movement such as eye deviation was reported in 17% of cases.^1^, ^2^, ^10^, ^11^, ^12^, ^13^ Movement disorder including hyperkinetic movements was identified in one patient.^14^ Approximately 56% of patients affected with PLP‐dependent epilepsy suffer developmental delay/intellectual disability.

Moreover, microcephaly was described in five cases.^15^, ^16^, ^17^, ^18^ Autistic spectrum disorders and attention‐deficit/hyperactivity disorder (ADHD) were observed in three patients.^7^, ^13^, ^19^

#### 3.1.2 PLP/PN clinical seizure responsiveness

PN was tried in most of the patients earlier considering pyridoxine‐dependent epilepsy (PDE) as one of the causes of refractory neonatal seizures. PN has tried 67 patients with variable clinical response; 44% (n = 29) showed remarkable clinical response and continued on PN alone, and 36% (n = 24) there was no response. 20% (n = 14) in whom PN was tried initially showed some sort of initial response then relapse of the seizure later and/or partial response with breakthrough seizures while on PN, which prompted a trial of PLP.

PLP trial was the second therapeutic option after PN in neonatal refectory seizure, used in 36 patients. Responses to PLP varied; 61% (n = 22) showed a marked immediate clinical response with seizures cessation including one patient required combined PN/PLP and riboflavin therapy to achieve seizure cessation^20^ and another patient never experienced seizure as PLP was initiated prophylactically,^21^ 25% (n = 9) of the responsive patients developed an immediate adverse event either like apneia‐hypotonia prior to seizure cessation (n = 4) or developed status epileptics upon switching to PLP (n = 5) and 14% (n = 5) responded only after several days with gradual seizure control. We noted that all of the responsive patients became PLP‐dependent and dose adjustment was needed to manage sick days. Abnormal liver function was observed in four patients after long time of administration of PLP (two were on 50 mg/kg/day and two other patients were on100 mg/kg/day).^22^, ^23^, ^24^, ^25^ In spite of effective seizure control with PLP, approximately 56% of patients affected with PLP‐dependent epilepsy suffer developmental delay/intellectual disability.

#### 3.1.3 Brain imaging

In the surveyed literature, neuroimaging findings were described in 55 patients (63%) with PNPO deficiency. There were no specific imaging features identified in the brains of the reviewed patients. Different radiological findings were described; however, 34 patients (61.8%) had normal brain imaging.^7^, ^8^, ^9^, ^11^, ^12^, ^13^, ^16^, ^21^, ^26^, ^27^, ^28^, ^29^, ^30^, ^31^, ^32^, ^33^ Di Salvo et al reported three patients with a c.347G > A (p. Arg116Gln) mutation in the *PNPO* gene who had normal brain imaging. These patients had residual enzyme activity accompanied by later onset and a milder seizure phenotype than patients with total loss of PNPO function.^7^ One patient reported with homozygous for a c.283C > T; (p. R95C) mutation at *PNPO* gene who had a normal brain MRI initially at 1 month of age but his follow‐up imaging at 3 years showed severe diffuse atrophy.^11^ The most common abnormality revealed on brain imaging was diffuse atrophy, which was observed in eight patients (14.5%),^8^, ^9^, ^16^, ^20^ followed by ischemic changes and encephalomalacia in five patients (5.5%),^1^, ^13^, ^16^, ^17^ and delayed myelination and atrophy in another three patients.^18^, ^34^, ^35^ An additional two patients (2.5%) had diffuse or focal edema.^1^, ^10^ The remaining four patients were found to have venous sinus thrombosis, a small subdural hemorrhage, simplified pattern of gyrus and sulcus anatomy and nonspecific T2 signal in the periventricular white matter, which were probably not related to PNPO deficiency.^9^, ^33^, ^36^

#### 3.1.4 EEG features

The EEG features of patients with PNPO deficiency are variable. Burst‐suppression patterns are the most common feature, reported in approximately half of the cases, followed by multifocal spikes and sharp waves, and generalized spikes and waves discharge. Hypsarrhythmia has been reported in some cases. A minority have normal EEG. Table S3 shows details regarding the patients' EEG features.

### 3.2 Non‐neurological manifestations of PNPO deficiency

Most of the reported cases showed pre‐ and perinatal complications including pre‐maturity, fetal distress and intrauterine growth restriction (IUGR) with oligohydramnios.

Pre‐maturity was commonly observed in 50% of PNPO‐deficient patients and 58% of the pre‐mature cases suffered fetal distress. There were two detected cases of oligohydramnios^11^, ^35^ and two cases of IUGR.^11^, ^17^ Metabolic acidosis and/or lactic acidosis were reported in eight cases (Supplementary Table S2).

Hematological manifestations in the form of normocytic anemia, normochromic anemia or pancytopenia, ophthalmological changes if form of pigmentary retinopathy and gastrointestinal tract (GIT) manifestations including abdominal distension, constipation and feeding intolerance often occur in PNPO deficiency (Table 1).

**TABLE 1:** Manifestations concomitant with PNPO deficiency seizures

Concomitant non‐neurological disorders	No. of cases	References
Pre‐maturity	44	^1^, ^2^, ^8^, ^9^, ^10^, ^11^, ^13^, ^14^, ^15^, ^16^, ^17^, ^18^, ^20^, ^21^, ^25^, ^26^, ^27^, ^30^, ^34^, ^35^, ^36^, ^43^
Oligohydraminous	3	^10^, ^20^, ^35^
IUGR	2	^10^, ^17^
Lactic acidosis/metabolic acidosis	8	^1^, ^2^, ^15^, ^17^, ^20^, ^26^
Ophthalmological. Pigmentary retinopathy	2	^9^, ^14^
Anemia	9	^1^, ^9^, ^30^, ^36^
Poor weight gain	6	^1^, ^9^, ^17^
GIT features	–	–
Abdominal distention	2	^1^
Dilated bowel loops	1	^25^
Constipation	5	^9^
Feeding intolerance	1	^8^
Hypoglycemia	3	^17^, ^34^
Hypothyroidism	1	^9^
Cardiac disorders	2	^28^, ^36^

Abbreviations: IUGR, intrauterine growth restriction; PNPO, pyridoxamine‐5′‐phosphate oxidase.

### 3.3 Biochemical profiles in PNPO deficiency

The biochemical analysis protocols varied among the included studies. Notably, there was no available panel with specific biomarkers in the reviewed literature. The analytical testing in the majority of reports covered urine organic acids, with the main focus being on vanillactic acid (VLA), cerebrospinal fluid (CSF) neurotransmitters, and CSF and plasma amino acids.

Herein, we present the biochemical results for 59 cases provided by 22 reports (Supplementary Table S4). The biochemical tests used for diagnosis and their reference ranges vary among these studies. To allow a significant comparison, we only considered tests that were available for at least 10 patients. CSF 5‐HIAA and PLP were most frequently performed (>20 patients; Supplementary Figure S1). Elevation of urine VLA and decrease of CSF PLP were significant in the majority of patients, which make these measurements potential suggestive biomarkers for PNPO deficiency. Other tests are summarized in Table 2.

**TABLE 2:** Summary of the most significant biochemical profiles in PNPO deficiency biofluids. Results were acquired based on the reference range provided by each study and patients were not on PLP or PN treatments

Biofluid	Biomarker	Variables			Total number of patients	Rank
		Low (%)	Normal %	High %		
Urine	VLA	0	9	91	11	1
CSF	PLP	81	19	0	21	2
CSF	Glycine	6	12	80	16	3
CSF	Threonine	0	25	75	20	4
CSF	HVA	52	24	18	17	5
CSF	5‐HIAA	40	36	24	25	6
Plasma	Arginine	39	56	6	18	7
CSF	3‐OMD	0	63	37	19	8

Abbreviations: CSF, cerebrospinal fluid; HVA, homovanillic acid; 5HIAA, 5‐hydroxyindoleacetic acid; 3 OMD, 3‐ortho‐methyldopa; PLP, pyridoxal 5‐phosphate; PN, pyridoxine; PNPO, pyridoxamine‐5′‐phosphate oxidase; VLA, vanillactic acid.

### 3.4 Molecular aspect of PNPO deficiency

#### 3.4.1 Structural analysis and predicted effect of variants

**FIGURE 3:**
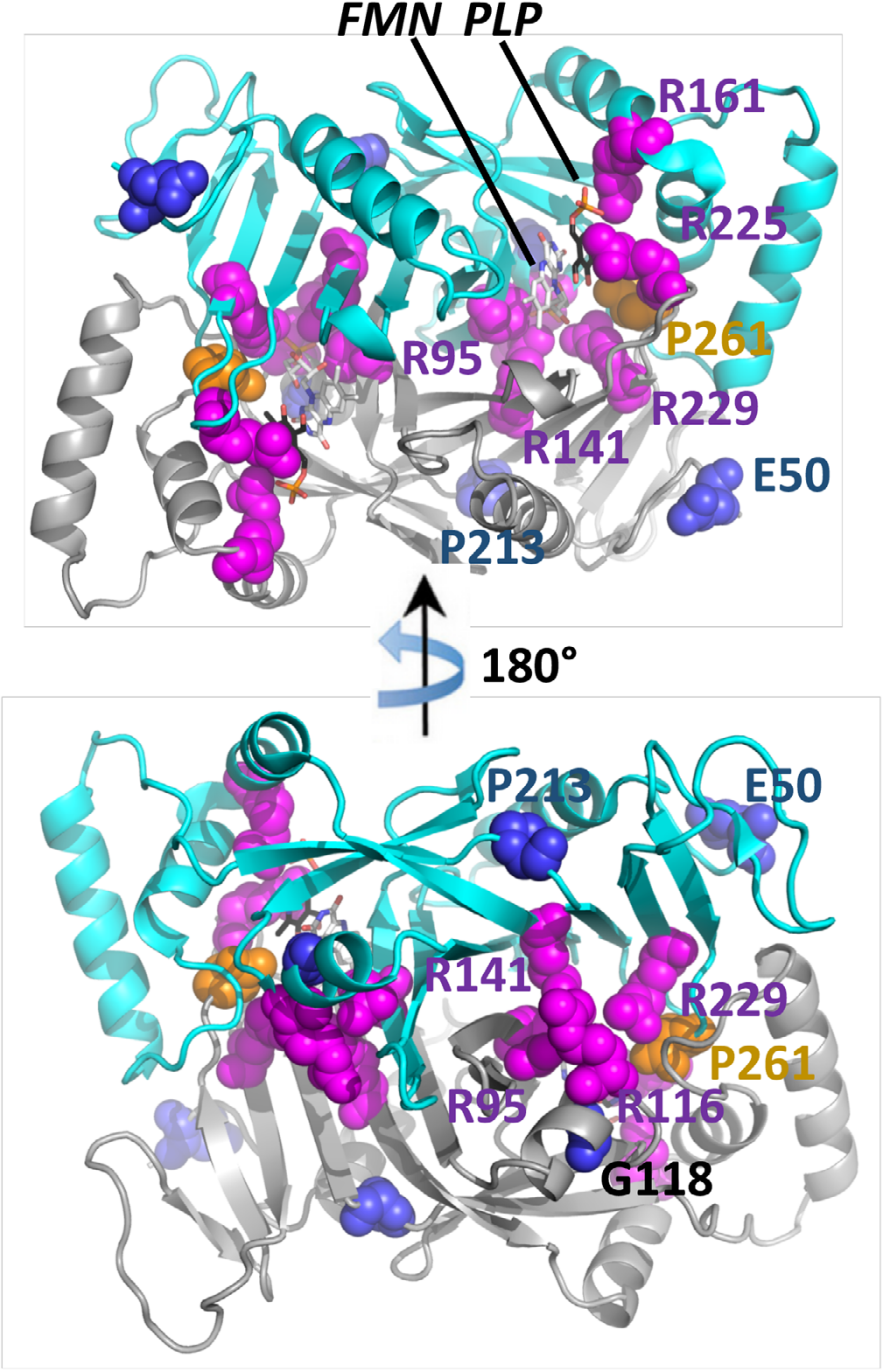
Overview of the reported point mutations. The crystal structure (PDB ID 1nrg) shows the post‐catalytic PLP (magenta) and FMN (dark lilac) as stick models. Point mutations are shown in sphere representation. Green coloration indicates that the mutation directly affects the binding and processing of the substrate and cofactor. Orange coloration indicates that the mutation decreases the protein's overall stability. FMN. flavin mononucleotide; PLP, pyridoxal 5′‐phosphate

Based on their position in the protein structure (Protein Data Bank (PDB) accession number 1nrg^37^ and type of mutation, PNPO variants can be separated into four categories. The largest category (here termed category I) contains mutations that directly affect the catalytic site and its capacity for ligand and cofactor binding (colored magenta in Figure 3 and Table 3). These mutations include R95C, R116Q, R141C, R161C, R225C/L/H, and R229W/Q. Strikingly, all these substitutions change a long and positively charged arginine into a polar or hydrophobic residue with different side chain dimensions. Accordingly, all of them impair the coordination of the negatively charged phosphate moieties of PLP or FMN, and sometimes have additional effects (eg, loss of π‐stacking interactions with PLP in the case of R225 mutations, or loss of inter‐chain ionic bonds for R116Q).

**TABLE 3:** Molecular analysis results shows the PLP and PN responsiveness as well as the clinical outcome of each variant

Effect	Mutation	Clinical observations	Molecular basis	Reference
Normal	D33V	Normal neurodevelopmental outcome at 11 y	Non‐homologous substitution in disordered N‐terminal extension	^11^, ^21^, ^22^
	D33V	Normal neurodevelopmental outcome at 11 mo	Non‐homologous substitution in disordered N‐terminal extension	^28^
	D33V	Normal neurodevelopmental outcome at 7 y	Non‐homologous substitution in disordered N‐terminal extension	^21^
	R116Q	Normal neurodevelopmental outcome at 3 y and 7 mo	Destabilizing FMN, loss of inter‐molecular ionic bond with E143	^7^
	G118R	Normal neurodevelopmental outcome at 6 y	Severe destabilizing clashes with surrounding	^20^
	R141C	Normal IQ, recurrent breakthrough seizure and SE, seizure free at the age of 26 y for the last 4 y (cp hetero)	Loss of FMN phosphate coordination	^16^
	P213S	Normal neurodevelopmental outcome at 5 y	Loss of minor hydrophobic interactions	^21^
	R225H	Normal neurocognitive development at 3 y and 10 mo	Loss of PLP coordination	^16^
	R225H	Normal neuro‐cognitive outcome at 6 years and 10 months	Loss of PLP coordination	^16^
	R225C	Normal neurodevelopmental status, and improved axial tone	Loss of PLP coordination	^16^
Mild to Moderate	R116Q	Moderate GDD at 12 y	Destabilizing FMN, loss of inter‐molecular ionic bond with E143	^7^
	R116Q	Mild GDD at 6 y	Destabilizing FMN, loss of inter‐molecular ionic bond with E143	^7^
	G118R	GDD	Severe destabilizing clashes with surrounding	^9^
	G118R	GDD	Severe destabilizing clashes with surrounding	^9^
	G118R	Mild hypotonia and GDD at 28 mo	Severe destabilizing clashes with surrounding	^12^
	R141H	Normal growth and development patterns neurological at 4 mo	Loss of FMN phosphate coordination	^30^
	P150Rfs*27	Normal neurodevelopmental outcome at the age of 12 mo	Non‐functional fragment	^27^
	R161C	Normal neurodevelopmental outcome at the age of 14 mo	Loss of PLP phosphate coordination	^29^
	R225H	ASD	Loss of PLP coordination	^13^
	R225H	Normal neurocognitive development and seizure free at 1 y and 8 mo. Unsteady gait	Loss of PLP coordination	^16^
	R225H	Normal neurocognitive development and seizure free at 1 y and 5 mo. Unsteady gait	Loss of PLP coordination	^16^
	R225H	GDD, seizure free at 9 y and 9 mo	Loss of PLP coordination	^16^
	R225H	GDD, recurrent breakthrough seizure	Loss of PLP coordination	^16^
	R225L	GDD	Loss of PLP coordination	^9^
	R225L	GDD	Loss of PLP coordination	^9^
	R225L	GDD	Loss of PLP coordination	^9^
	R225L	GDD	Loss of PLP coordination	^9^
Severe	E50K	Severe DD, microcephaly, spasticity at 2 y	Loss of minor stabilizing interactions	^1^, ^15^
	E50K	Died at 6 wk	Loss of minor stabilizing interactions	^1^, ^15^
	R95C	Died at 7 mo	Loss of FMN phosphate coordination	^11^
	R95C	Severe psychomotor delay at 2 y and 5 mo	Loss of FMN phosphate coordination	^11^
	R95C	Died	Loss of FMN phosphate coordination	^11^
	R95C	Died	Loss of FMN phosphate coordination	^11^
	R95C	Severe psychomotor delay	Loss of FMN phosphate coordination	^11^
	R95C	Death at 47 DOL	Loss of FMN phosphate coordination	^36^
	R95C	Died at 31 d	Loss of FMN phosphate coordination	^26^
	R95C	Psychomotor development was severely delayed and epileptic phenomena were almost constantly present at 3 y old	Loss of FMN phosphate coordination	^26^
	P150Rfs*27/R161G (cp heteroz)	Neurodevelopmental outcome was poor with severe developmental delay, cortical visual impairment and autistic features	Non‐functional fragment/loss of PLP phosphate coordination	^19^
	A174X	Died at 48 d of life (DOL)	Non‐functional fragment	^35^
	R225H	Died at 14 mo	Loss of PLP coordination	^18^
	R225H	Psychomotor retardation with autonomous gait at 2.5 y and a slight intellectual disability	Loss of PLP coordination	^30^
	R225H	Severe GDD, spastic tetraplegia, progressive microcephaly, recurrent breakthrough seizure at 8 y	Loss of PLP coordination	^16^
	R225H	Severe GDD, spastic tetraplegia, progressive microcephaly, recurrent breakthrough seizure while on PN	Loss of PLP coordination	^16^
	R225C	Global psychomotor delay at the age of 3 y	Loss of PLP coordination	^17^
	R229Q	Normal head growth, pigmentary retinopathy, diffuse hypotonia, and hyperkinetic movements	Loss of FMN phosphate coordination	^14^
	R229Q	Hemiparesis at 21 mo after middle cerebral artery ischemic stroke	Loss of FMN phosphate coordination	^13^
	R229W	Died at 21 DOL	Loss of FMN phosphate coordination	^1^, ^2^
	R229W	Died on first DOL	Loss of FMN phosphate coordination	^1^, ^2^
	R229W	Died at 6 mo	Loss of FMN phosphate coordination	^1^, ^2^
	R229W	Died at 17 DOL	Loss of FMN phosphate coordination	^1^, ^2^
	R229W	Died at 19 DOL	Loss of FMN phosphate coordination	^1^, ^2^
	X262Q	Died at 23 DOL	Additional residues disrupt ligand binding and 3D‐fold	^1^
	X262Q	Died at 15 DOL	Additional residues disrupt ligand binding and 3D‐fold	^1^

Abbreviations: DD, developmental delay; FMN. flavin mononucleotide; PLP, pyridoxal 5′‐phosphate; PN, pyridoxine.

The second category, category II, contains mutations that affect the fold and stability of the protein because of a non‐conservative side chain substitution (blue in Figure 3 and Table 3). Their effects range from a mild loss of stabilizing surface interactions (E50K) and loss of hydrophobic interactions (P213S) to severely destabilizing clashes (G118Q/R).

Category III is constituted by variants causing residue deletion (S93S_A94Ldel), pre‐mature stop codons (A174X), loss of stop codons (X262Q) and frame shifts combined with loss of subsequent protein sequences (L83W_fs*17, P150R_fs*27). In the compact structure of the PNPO dimer, a significant loss of residues would severely compromise the protein's structure and function. Hence, the truncating mutations are expected to lead to non‐functional proteins. PNPO has the particularity that its C‐terminus folds back inside the protein and terminates just before the substrate‐binding pocket. Accordingly, the loss of stop codon would disrupt both, ligand binding and protein fold (orange in Figure 3).

D33V is in a human‐specific N‐terminal extension and does not affect the 3D structure or catalytic function of the protein, placing it in category IV.^38^ D33V might mildly affect the (unknown) function of the N‐terminal extension.

#### 3.4.2 Correlation between molecular effects and clinical outcomes

Our overview reveals a striking lack of correlation between the reported clinical effects of PNPO variants and the molecular effects they have on the protein structure and function (Table 3). Some individuals showed apparently normal phenotypes, despite encoding for a clearly deleterious variant (eg, R116Q, G118R or P150R_fs*27), whereas others experienced most severe effects for a substitution that is expected to only mildly affect the protein's function (E50K). Moreover, the same variant may cause no or only mild effects in some individuals, but severely affect others (eg, R225H). However, the most severe complications appeared to be caused by category I and III variants. In particular, mutations of R95 and R229 were only found in the severe cases, and four out of five category III variants were ranked as “severe.” The unexpected severity of E50K (category II) appears not justified by its mildly destabilizing function and might indicate supplementary, unidentified human‐specific roles of this residue.

### 3.5 PNPO deficiency outcome

Finally, we assessed factors that impact the outcomes of PNPO deficiency based on cognitive function and/or developmental assessment. Pre‐maturity, fetal distress, seizure onset and initiation of PLP therapy have been documented and correlated with outcome variables including death, developmental delay and normal development (Supplementary Table S2).

**FIGURE 4:**
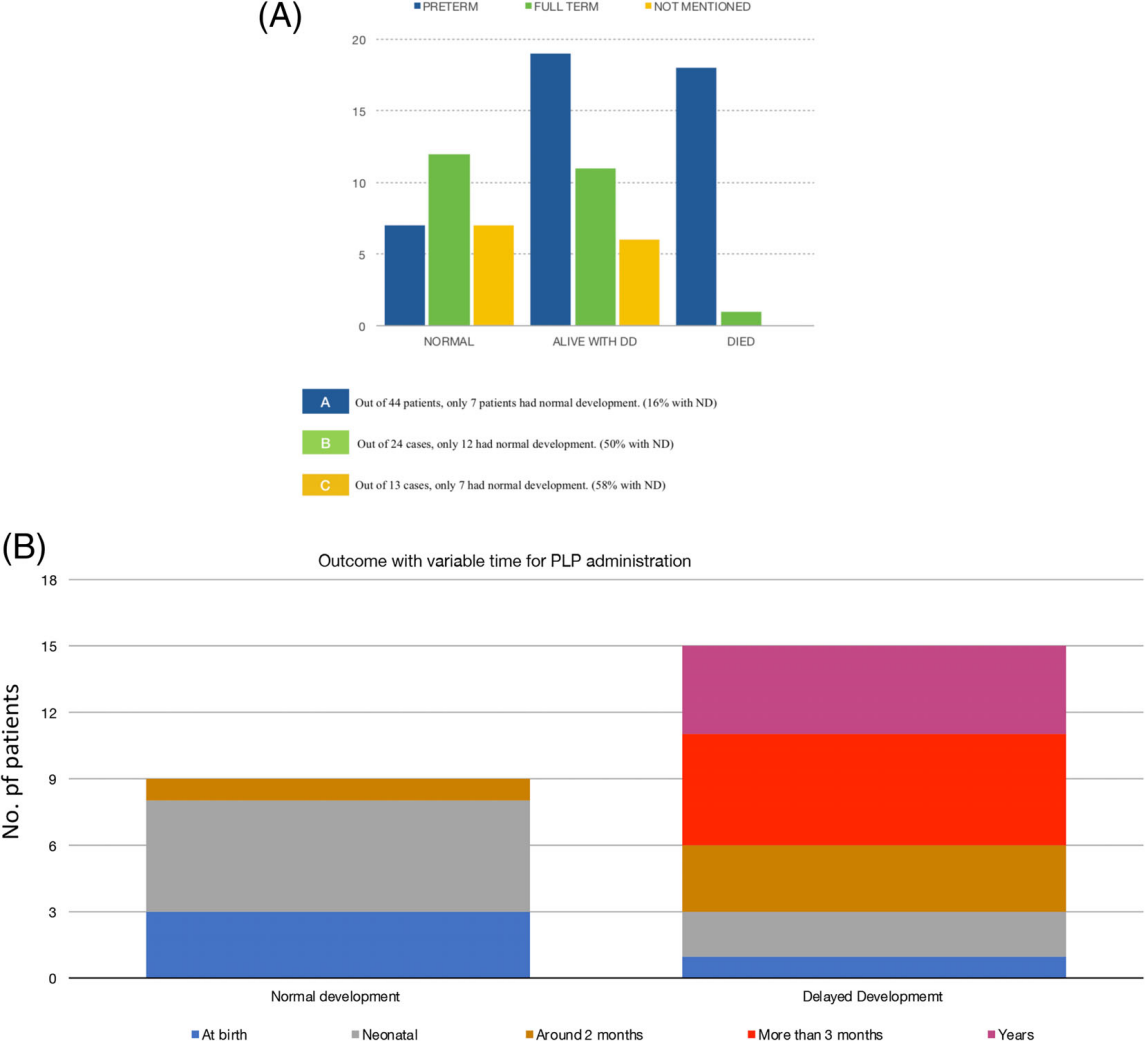
A, The impact of pre‐maturity on clinical outcomes, including death and developmental delay (DD). B, Variable outcomes with variable age of PLP administration. The earlier PLP was introduced as a therapy, the better the prognosis was reported. PLP, pyridoxal 5′‐phosphate

To evaluate the impact of pre‐maturity, we studied all 87 cases based on gestational age at birth. Pre‐maturity of birth was observed in the majority of patients (n = 44); 24 of them had shown fetal distress during the neonatal period. Only seven patients developed normally given their pre‐maturity, while 37 patients either died or showed variable degrees of developmental delay. We have categorized patients based on the maturity and outcome (Figure 4A). Categories A, B and C represent the percentage of normal development within full term patients, pre‐mature patients and rest of patients, respectively. The apparent high correlation between pre‐maturity and unfavorable outcome could be caused by complications due to pre‐maturity itself and/or by the earlier onset of the disease.

We explored seizure onset and time for diagnosis and treatment as factors for the outcome. Seizures generally manifest early after birth, commonly during the first days of life. Almost more than half of patients (59%) developed seizures within their first day of life; 50% of them showed variable developmental delay, 25% died and the others developed normally.

Various clinical courses and outcomes were reported for patient treatment with PN and/or PLP. Fourteen cases lacked information about the treatment strategy. Among the 87 patients who received PN treatment (n = 67) in this review, 36% of the patients showed a negative response to PN, and 64% of the patients showed variable degrees of response; all of them survived, but most of them had various degrees of developmental delay. Only 16 patients who responded to PN as a main treatment developed normally; six of them were full term. Of the 19 patients who died, all of them are pre‐mature newborns and they did not respond to PN, one only showed an initial response to PN, and neither of them received PLP treatment.

PLP trial was introduced for 36 patients where it showed a dramatic response in 61% (n = 22) of the patients including 36% (n = 8) of them had not responded to PN as their initial therapy and 4% (n = 1) of them started PLP since birth prophylactically. We have documented developmental outcome and the time of PLP initiation in the surveyed literatures for 24 patients. We noticed that the earlier PLP was introduced as a therapy, the better was the prognosis (Figure 4B). Normal development was found in nine cases where PLP therapy was started as early as possible. Four of them were full term. The remaining 15 cases survived but had variable degrees of developmental delay, and some patients suffered hypotonia, microcephaly or increased tone in all four limbs. In majority of those cases, PLP was introduced late (Figure 4B).

## 4 CONCLUSION

Herein we provided a comprehensive review of the clinical, biochemical and molecular observations reported for PNPO deficiency. Our survey shows that the phenotypic spectrum of PNPO deficiency is wide, including a multitude of neurological and systemic manifestations. Its characteristic clinical feature is refractory seizures during the first year of life. On a molecular level, most of the PNPO variants either affect substrate and cofactor binding and processing, or weaken/disrupt the 3‐dimensional structure of PNPO. The molecular effect generally correlates only in some cases with the severity of the symptoms. For example, mutations that disrupt the protein's catalytic function produce a more pronounced clinical outcome than mutations that are only mildly destabilizing. However, in many cases the clinical outcomes for the same PNPO variant can vary significantly between patients, highlighting the importance of the timing and nature of treatment administered.

Indeed, we noted that pre‐maturity, delayed therapeutic intervention and an earlier onset of clinical seizures correlated with a poorer neurocognitive outcome. Given the amenability of PNPO‐dependent epilepsy to treatment with PLP and PN therapy for optimal seizure control and favorable developmental outcomes, early diagnosis is essential. Therefore, emphasis should be placed on early testing and disease detection, especially in cases of a suspected family history indicating PNPO deficiency. Furthermore, prenatal supplementation of suspected cases should be considered in the presence of certain conditions, such as prenatal seizures or an index case in the family. Prenatal supplementation in PNPO deficiency has not been indicated yet, particularly as a therapy for cessation or prevention of prenatal seizures and/or fetal distress.

Although several biochemical changes indicate multiple enzyme defects (Table 2), there is currently no specific biomarker for PNPO deficiency. However, we noted that the combination of elevated urinary VLA, CSF glycine and decreasing CSF PLP might be a suggestive profile that could be used for infants presenting with unexplained refractory seizures and motivate a *PNPO* molecular analysis. Measuring PNPO enzyme activity through a rapid LC‐MS/MS‐based dried blood spot assay has also been proposed for rapid diagnosis of PNPO deficiency.^39^

It is important to differentiate between PNPO deficiency and other B6‐dependent epilepsies, including alpha‐aminoadipic semialdehyde dehydrogenase (ALDH7A1) deficiency and pyridoxal phosphate‐binding protein (PLPBP) deficiency. Reliable biomarkers for ALDH7A1 disorder exist, and include an elevated urinary AASA/creatinine ratio, elevated plasma pipecolic acid, 6‐oxo‐pipecolate,^40^ and a characteristic peak in the analysis of CSF monoamine metabolites by HPLC.^41^ PLPBP deficiency, however, is another B6‐dependent epilepsy associated with low‐CSF‐PLP akin to PNPO deficiency. Hence, diagnosis of different B6‐dependent epilepsies is best established by the identification of biallelic pathogenic variants in the *ALDH7A1*, *PNPO* and *PLPBP* genes.

Four cases of PNPO deficiency were presented with liver cirrhosis or abnormal liver function test after receiving PLP treatment. Although abnormal liver function might expand the phenotypic spectrum of PNPO deficiency, the PLP administration is probably the cause of liver impairment. Schmitt et al. reported a 2 years and 6 months old boy with PNPO deficiency who developed an abnormal liver function test after escalating a PLP dose to 100 mg/kg/day and it had to be reduced to 53 mg/kg/day.^22^ Sudarsanam et al have postulated that liver dysfunction in their patient was due to a high dose of administered PLP (100 mg/kg/day).^23^ Although reduction of the PLP dose and frequency resulted in substantial reduction in the liver transaminases, episodes of uncontrolled seizures and encephalopathy required high doses of PLP, which negatively affected the liver function.^23^ Porri et al has also reported a mild elevation in alanine aminotransferase and aspartate aminotransferase levels on a PNPO‐deficient patient treated with 50 mg/kg/day of PLP.^25^ Later, Coman et al described a 4 years old boy with PNPO deficiency in whom liver cirrhosis has been showed while receiving a 50 mg/kg/day of PLP.^24^ Last two cases have never received “high dose” of PLP, rather a dosage range of 30 to 50 mg/kg/day.^24^, ^25^ In all PNPO‐deficient patients, liver derangement occurred after long‐term administration of PLP with substantial improvement after dose adjustment which indicates that liver toxicity is probably related to PLP administration and should be carefully monitored.^22^, ^23^, ^24^, ^25^ In addition, a previously reported liver toxicity case secondary to high‐dose PLP for treating homocystinuria was documented by Yoshida et al supported further this hypothesis.^42^ Collectively, these reports highlight the possibility of PLP‐related liver dysfunction in PNPO‐deficient patient, and hence surveillance for evidence of liver cirrhosis should be part of management of PNPO‐deficient patients receiving PLP.

We conclude that early detection of PNPO deficiency combined with early PLP treatment is key to optimizing the clinical outcome. While newborn screening is useful for the early detection of some diseases, it might not be feasible in PNPO deficiency due to the absence of sensitive biomarkers. However, we identified suggestive biochemical profiles in the literature that should motivate a definitive molecular diagnosis of *PNPO* gene variants, especially in cases of a suspected family history indicating PNPO deficiency.

## CONFLICT OF INTEREST

The authors declare no potential conflict of interest.

### PEER REVIEW

The peer review history for this article is available at https://publons.com/publon/10.1111/cge.13843.

## Supporting information


**Figure S1** The most common biochemical tests requested for PNPO diagnosis (2002‐2020)

**Table S1** The list of genes coding for PLP‐dependent enzymes.

**Table S2** Patient information included patient age, gender, seizure onset, family history, parents' consanguinity, pregnancy pre‐maturity (gestational age), Apgar score, condition at birth, status of failure to thrive, neurological changes, electroencephalogram (EEG) changes, MRI tests, the results for molecular and biochemical analysis and responsiveness to antiepileptic therapy, pyridoxine therapy, and PLP trial.

**Table S3** EEG changes in PNPO deficiency.

**Table S4** comprehensive biochemical profile for each reported case.

## Data Availability

The data that supports the findings of this study are available in the supplementary material of this article
