# Supplementary material for: Phenotypic and molecular spectrum of pyridoxamine‐5′‐phosphate oxidase deficiency: A scoping review of 87 cases of pyridoxamine‐5′‐phosphate oxidase deficiency
Source: Clin Genet. 2020 Sep 16;99(1):99–110. doi: 10.1111/cge.13843 (PMC7820968; doi:10.1111/cge.13843)
Supplement: Supplementary file 1 — Figure S1 The most common biochemical tests requested for PNPO diagnosis (2002‐2020) [file CGE-99-99-s001.docx]

**Supplementary Figure 1**: The most common biochemical tests requested for PNPO diagnosis (2002-2020)
