# Supplementary material for: Phenotypic and molecular spectrum of pyridoxamine‐5′‐phosphate oxidase deficiency: A scoping review of 87 cases of pyridoxamine‐5′‐phosphate oxidase deficiency
Source: Clin Genet. 2020 Sep 16;99(1):99–110. doi: 10.1111/cge.13843 (PMC7820968; doi:10.1111/cge.13843)
Supplement: Supplementary file 2 — Table S1 The list of genes coding for PLP‐dependent enzymes. [file CGE-99-99-s002.docx]

|  |  |  |
| --- | --- | --- |
| **GLDC** | **Glycine decarboxylase** | Biased expression in kidney (RPKM 25.8), placenta (RPKM 17.4) liver, brain (RPKM 7.03 +/- 4.17) |
| **SHMT1** | **Serine hydroxymethyltransferase 1** | Kidney liver , fat , duodenum |
| **SHMT2** | **Serine hydroxymethyltransferase 2** | Ubiquitous expression in liver (RPKM 35.4), lymph node (RPKM 21.8) appendix, duodenum, brain (RPKM 10.5 +/- 6.67) |
| **GOT1** | **Glutamic-oxaloacetic transaminase 1** | Broad expression in heart (RPKM 196.1), liver (RPKM 87.7), ......................Brain (RPKM 67.4 +/- 26.5) |
| **GOT1L1** | **Glutamic-oxaloacetic transaminase 1 like 1** | Testis only exclusive expression |
| **GPT** | **Glutamic--pyruvic transaminase** | Liver, Kidney, fat and colon |
| **GPT2** | **Glutamic--pyruvic transaminase 2** | Broad expression in fat (RPKM 41.2), pancreas (RPKM 29.0), Liver, esophagus, prostate, .........Brain (RPKM 15.2 +/- 3.1 ) |
| **TAT** | **Tyrosine aminotransferas** | Restricted expression toward liver |
| **CCBL1/KYAT1** | **Kynurenine aminotransferase 1** | Ubiquitous expression in brain (RPKM 2.6), skin (RPKM 1.9 |
| **CCBL2** | **Kynurenine aminotransferase 3** | Ubiquitous expression in thyroid (RPKM 13.7), esophagus (RPKM 12.5), fat, brain (RPKM 7.08 +/- 2.11) |
| **OAT** | **Ornithine aminotransferase** | Broad expression in small intestine (RPKM 198.3), duodenum (RPKM 185.5) , adrenal, brain (RPKM 32.09 +/- 3.9) |
| **ABAT** | **4-aminobutyrate aminotransferase** | Biased expression in liver (RPKM 93.2), kidney (RPKM 61.6), brain (RPKM 53.8 +/- 23.9)<https://www.ncbi.nlm.nih.gov/gene/> 18#gene-expression |
| **AADAT** | **Aminoadipate aminotransferase** | Broad expression in liver (RPKM 11.4), prostate (RPKM 6.3) , endometrium, brain 3.73 +/- 1.01 |
| **BCAT2** | **Branched chain amino acid transaminase 2** | Ubiquitous expression in urinary bladder (RPKM 16.4), adrenal (RPKM 15.6), kidney, heart, colon, ......................., brain RPKM 2.7 +/- 0.4 |
| **AGXT2** | **Alanine--glyoxylate aminotransferase 2** | Mainly Kidney, Liver |
| **ETNPPL/ AGXT2L1** | **Ethanolamine-phosphate phospholyase** | Biased expression in liver , brain |
| **AGXT2L2** | **5-phosphohydroxy-L-lysine phospho-lyase** | small intestine, spleen, duodenum, appendix, ..................., brain |
| **AGXT** | **Alanine--glyoxylate and serine--pyruvate aminotransferase** | Liver only |
| **PSAT1** | **Phosphoserine aminotransferase 1** | Biased expression in liver (RPKM 37.5), brain (RPKM 36.7) |
| **NFS1** | **NFS1 Cysteine desulfurase, mitochondrial** | Ubiquitous expression in kidney (RPKM 16.1), testis (RPKM 12.1), adrenal, liver and brain 8.2 +/- 0.6 |
| **MOCOS** | **Molybdenum cofactor sulfurase** | very low amount in brain |
| **KYNU** | **Kynureninase** | liver, urinary bladder, appendix, testis,..........., brain (very low expression) Chr. 2 |
| **GAD1** | **Glutamate decarboxylase 1** | Biased expression in brain (RPKM 27.1) MAINLY Brain and kidney(lesser extent) Chr. 2 |
| **GAD2** | **Glutamate decarboxylase 2** | Restricted expression toward brain (RPKM 7.7). Chr. 10 |
| **ODC1** | **Ornithine decarboxylase 1** | testis, bone marrow, lymph node, prostate, appendix, .....................Brain |
| **HDC** | **Histidine decarboxylase** | Gall bladder stomach ,........................, to a lesser extent in brain |
| **DDC** | **Dopa decarboxylase** | a trivial expression in brain |
| **GADL1** | **Glutamate decarboxylase like 1** | Prostate , kidney , oesophagus, kidney |
| **CSAD** | **Cysteine sulfinic acid decarboxylase** | prostate , fat, endometrium, skin, ......................, Brain |
| **SGPL1** | **Sphingosine-1-phosphate lyase 1** | UB, Skin, Intestine, appendix, .................., brain |
| **CBS** | **Cystathionine beta-synthase** | Biased expression in liver, brain RPKM 11.6 |
| **THNSL1** | **Threonine synthase like 1** | Ubiquitous expression in Liver, thyroid , kidney , adrenal, brain |
| **THNSL2** | **Threonine synthase like 2** | Kidney, thyroid, adrenal, fat, duodenum, .............., brain |
| **SDS** | **Serine dehydratase** | Biased expression in liver (RPKM 130.0) and brain( low expression ) |
| **SDSL** | **Serine dehydratase like** | Kidney, thyroid, liver, adrenal, duodenum ,colon, testis, brain |
| **CTH** | **Cystathionine gamma-lyase** | Liver, kidney, adrenal, thyroid, colon, duodenum, brain |
| **ACCS** | **1-aminocyclopropane-1-carboxylate synthase homolog (inactive)** | Spleen, gall bladder, endometrium , adrenal ,.......................... brain (low expression) |
| **ACCSL** | **1-aminocyclopropane-1-carboxylate synthase homolog (inactive) like** | Low expression observed in reference data, testis, skin, brain( RPKM: 0,05+_0,02 , endometrium |
| **SCLY** | **Selenocysteine lyase** | kidney, testis, liver, duodenum, .........., brain (2,03+-.30 |
| **SRR** | **Serine racemase** | Ubiquitous expression in brain (RPKM 6.5), testis |
| **ADC/AZIN2** | **Antizyme inhibitor 2** | ????/PROVISIONAL gene |
| **AZIN1** | **Antizyme inhibitor 1** | Ubiquitous expression in brain (RPKM 50.0), bone marrow (RPKM 45.4 |
| **SEPSECS** | **O-phosphoseryl-tRNA(Sec) selenium transferase** | Ubiquitous expression in liver (RPKM 7.2), duodenum, small intestine, kidney,.... Brain (RPKM: 1,64 +0,5) |
| **PROSC** | **Pyridoxal phosphate binding protein** | Ubiquitous expression in kidney (RPKM 12.8), colon (RPKM 10.6), .... Brain (RPKM 8.3 +/-1.53 ) |
| **PDXDC1** | **Pyridoxal dependent decarboxylase domain containing 1** | Ubiquitous expression in testis (RPKM 42.5), duodenum (RPKM 33.1),................... Brain (RPKM 8.04 + 0.78 ) |
| **PYGB** | **Glycogen phosphorylase B** | Broad expression in colon (RPKM 67.8), heart (RPKM 66.8), esophagus, prostate, Brain (RPKM 39.71+/-07.6) *The protein encoded by this gene is a glycogen phosphorylase found predominantly in the brain. |
| **PYGM** | **Glycogen phosphorylase, muscle associated** | Biased expression in esophagus (RPKM 41.2), prostate, ..........,Brain (RPKM 6.11+/- 4.00) |
| **PYGL** | **Glycogen phosphorylase L** | Broad expression in Fat, BM, Placenta, liver, UB, .................... Brain (RPKM 3.7+/-2.27) |
| **SPTLC1** | **Serine palmitoyltransferase long chain base subunit 1** | Ubiquitous expression in thyroid (RPKM 21.8), esophagus (RPKM 19.8, ....................... Brain (RPKM 13.2 +/- 1.43) |
| **SPTLC2** | **Serine palmitoyltransferase long chain base subunit 2** | Ubiquitous expression in esophagus (RPKM 11.7), colon (RPKM 11.4), ............... Brain (RPKM 9.54 +/- 0.9 ) |
| **SPTLC3** | **Serine palmitoyltransferase long chain base subunit 3** | Broad expression in placenta (RPKM 7.5), thyroid (RPKM 7.0), ................ Brain (RPKM 0.69 +/-0.17) |
| **ALAS2** | **5'-aminolevulinate synthase 2** | Restricted expression toward bone marrow |
| **ALAS1** | **5'-aminolevulinate synthase 1** | Biased expression in adrenal (RPKM 297.6), liver (RPKM 180.6), ...................................... Brain (RPKM 13.25 +/-2.07) |
| **GCAT** | **Glycine C-acetyltransferase** | Ubiquitous expression in pancreas (RPKM 12.0), .................. Brain (RPKM 3.2 +/- 0.3 ) |
