# Supplementary material for: Phenotypic and molecular spectrum of pyridoxamine‐5′‐phosphate oxidase deficiency: A scoping review of 87 cases of pyridoxamine‐5′‐phosphate oxidase deficiency
Source: Clin Genet. 2020 Sep 16;99(1):99–110. doi: 10.1111/cge.13843 (PMC7820968; doi:10.1111/cge.13843)
Supplement: Supplementary file 4 — Table S3 EEG changes in PNPO deficiency. [file CGE-99-99-s004.docx]

| Ref. (68 patients) | EEG findings |
| --- | --- |
| Angeles et al. 2008 (1 patient) | Consistent with severe myoclonic encephalopathy and burst-suppression pattern (BSP) |
| Bagci et al. 2008 (3 patients) | Patient 1: N/A  Patient 2: BSP  Patient 3: Interictal EEG Normal, one EEG showed subclinical left temporal spike wave discharges |
| Bräutigam et al. 2002 (2 patients were twins) | BSP |
| Clayton et al 2003 | N/A |
| Di Salvo et al. 2017 (3 patients) | Patient 1: Interictal sleep EEG showed: Bilateral spikes, and slow waves in the fronto-temporal regions. Diffuse spikes and polyspikes and wave discharges with posterior predominance.  Patient 2: Initial EEG showed: Slow background activity with generalized spike and wave discharges. Repeat EEG showed slow background activity and multiple spike-and-wave discharges lasting up to 5–6 s  Patient3: Slowing of background activity and an association of focal and generalized spike and wave discharges |
| Goyal et al 2013 (4 patients) | Patient 1: Continuous generalized delta slowing interrupted by generalized bursts of multiple spikes  Patient 2: Evolution from hypsarrhythmia to generalized slow spike and wave discharges  Patient 3: NA Patient 4: EEG correlation with the multifocal clonic events |
| Guerriero et al. 2017  (6 patients) | EEG background abnormalities were diverse and included lack of organization and state transitioning (two), discontinuity (two), and multifocal and generalized epileptiform discharges (six) |
| Guerin et al. 2014 (1 patient) | Slow background and bilateral independent frequent sharp waves in the centrotemporal areas |
| Hatch et al. 2016 (4 patients) | Patient 1: BSP  Patient 2: An EEG at 24 h of age was normal. At 3 months of age, EEG showed periods of focal slowing with sharp waves overlaid, in a posterior distribution  Patient 3: An EEG at 4 weeks of age was normal. At 6 weeks of age, there were periods of slowing and frequent intermixed multifocal sharp waves.  8 weeks of age: when seizures had changed to include tonic events with vocalization, showed periods of electro decrement with ongoing multifocal epileptiform discharges.  Patient 4: Normal |
| Hoffman et al. 2007 (6 patients) | Patient 2: BSP  Patient 3: Several long-term EEGs were normal, even during the spasms. Only one EEG showed subclinical left temporal spike wave discharges  Patient 4: BSP |
| Jaeger et al. 2016 ( 1 patient) | BSP and centrally located rhythmic sharp and slow waves |
| Jorge García-ezquiaga et al. 2019 | N/A |
| Levtova et al. 2015 (1 patient) | Slow background and electrodecremental patterns without hypsarrhythmia |
| Lloreda-García et al. 2017  (1 patient) | BSP |
| Lugli et al. 2019 (1 patient) | BSP and generalized high-voltage poly spikes and slow-wave discharges |
| Meng Kuo et al. 2002 (1 patient) | Diffuse cortical dysfunction with paroxysmal multifocal sharp waves over bilateral parietal areas |
| Mills et al 2005 (5 patients) | Patient G6: BSP  Patient J2: BSP  Patient K1: Consistent with severe myoclonic encephalopathy Patient K2: BSP |
| Mohanlal et al. 2020  **(1 patient)** | Hypsarrhythmia later evolving into multifocal spike-and-slow wave discharges |
| Morad et al. 2008 (1 patient) | N/A |
| Phillip et al. 2012 (1 patient) | Bitemporal sharp discharges and episodic background suppression without clinical symptoms |
| Plecko et al. 2014 (11 patients) | Four patients: Discontinuous pattern   - Four patients: BSP - One patient: Flat tracing, then BSP - Two patients: N/A |
| Porri et al. 2014 (1 patient) | BSP with synchronized bursts of bilateral moderate-amplitude spike-and-wave discharges |
| Riikonen et al. 2015 | N/A |
| Veerapandiyan et al. 2011  (1 patient) | Patient 1: BSP with bilateral, independent multifocal sharp waves  Patient 2: Slow background and multifocal spike/slow wave discharges |
| Veeravigrom et al. 2015  (1 patient) | BSP |
| Ware et al. 2013 (2 patients) | Patient 1: Normal Patient 2: Slow background and multifocal discharges |
| Xue et al. 2017 (4 patients) | One patient: Normal  One patient: Hypsarrhythmia in association with multifocal discharges  Three patients: Multifocal discharges |
| Alghamdi et al. 2019  **(2 patient)** | Patient 1: N/A  Patient 2: BSP at the age of one week. At the age of 6 months demonstrates multifocal asynchronous, frequent spikes and polyspikes more over the right temporal and central areas |

BSP: Burst-suppression Pattern
